# *BRCA1* and *BRCA2* rearrangements in Brazilian individuals with Hereditary Breast and Ovarian Cancer Syndrome

**DOI:** 10.1590/1678-4685-GMB-2014-0350

**Published:** 2016

**Authors:** Ingrid Petroni Ewald, Silvia Liliana Cossio, Edenir Inez Palmero, Manuela Pinheiro, Ivana Lucia de Oliveira Nascimento, Taisa Manuela Bonfim Machado, Kiyoko Abe Sandes, Betânia Toralles, Bernardo Garicochea, Patricia Izetti, Maria Luiza Saraiva Pereira, Hugo Bock, Fernando Regla Vargas, Miguel Ângelo Martins Moreira, Ana Peixoto, Manuel R. Teixeira, Patricia Ashton-Prolla

**Affiliations:** 1 Laboratório de Medicina Genômica, Hospital de Clínicas de Porto Alegre (HCPA), Porto Alegre, RS, Brazil.; 2 Programa de Pós-Graduação em Medicina: Ciências Médicas, Universidade Federal do Rio Grande do Sul (UFRGS), Porto Alegre, RS, Brazil.; 3 Centro de Pesquisa em Oncologia Molecular - Hospital do Câncer de Barretos, Barretos, SP, Brazil; 4 Departamento de Genética, Instituto Português de Oncologia do Porto, Porto, Portugal.; 5 Laboratório de Imunologia e Biologia Molecular, Instituto de Ciências da Saúde (ICS), Universidade Federal da Bahia (UFBA), Salvador, BA, Brazil.; 6 Departamento de Pediatria, Universidade Federal da Bahia (UFBA), Salvador, BA, Brazil.; 7 Centro de Oncologia, Hospital Sírio Libanês, São Paulo, SP, Brazil.; 8 Programa de Pós Graduação em Genética e Biologia Molecular, Universidade Federal do Rio Grande do Sul (UFRGS), Porto Alegre, RS, Brazil.; 9 Laboratório de Identificação Genética, Hospital de Clínicas de Porto Alegre (HCPA), Porto Alegre, RS, Brazil.; 10 Serviço de Genética Médica, Hospital de Clínicas de Porto Alegre (HCPA), Porto Alegre, RS, Brazil.; 11 Departamento de Bioquímica, Universidade Federal do Rio Grande do Sul (UFRGS), Porto Alegre, RS, Brazil.; 12 Divisão de Genética, Instituto Nacional de Câncer (INCA), Rio de Janeiro, RJ, Brazil; 13 Instituto Nacional de Genética Médica Populacional (INAGEMP), Hospital de Clínicas de Porto Alegre (HCPA), Porto Alegre, RS, Brazil.; 14 Laboratório de Genômica Funcional e Bioinformática, Instituto Oswaldo Cruz (IOC-FIOCRUZ), Rio de Janeiro, RJ, Brazil.; 15 Departamento de Genética e Biologia Molecular, Centro de Ciências Biológicas e da Saúde, Universidade Federal do Estado do Rio de Janeiro (UFRJ), Rio de Janeiro, RJ, Brazil.; 16 Instituto de Ciências Biomédicas Abel Salazar (ICBAS), Porto, Portugal.

**Keywords:** Breast cancer, Hereditary Breast and Ovarian Cancer syndrome, gene rearrangements, *BRCA* gene

## Abstract

Approximately 5-10% of breast cancers are caused by germline mutations in high penetrance predisposition genes. Among these, *BRCA1* and *BRCA2*, which are associated with the Hereditary Breast and Ovarian Cancer (HBOC) syndrome, are the most frequently affected genes. Recent studies confirm that gene rearrangements, especially in *BRCA1*, are responsible for a significant proportion of mutations in certain populations. In this study we determined the prevalence of *BRCA* rearrangements in 145 unrelated Brazilian individuals at risk for HBOC syndrome who had not been previously tested for *BRCA* mutations. Using Multiplex Ligation-dependent Probe Amplification (MLPA) and a specific PCR-based protocol to identify a Portuguese founder *BRCA2* mutation, we identified two (1,4%) individuals with germline *BRCA1* rearrangements (c.547+240_5193+178del and c.4675+467_5075-990del) and three probands with the c.156_157insAlu founder *BRCA2* rearrangement. Furthermore, two families with false positive MLPA results were shown to carry a deleterious point mutation at the probe binding site. This study comprises the largest Brazilian series of HBOC families tested for *BRCA1* and *BRCA2* rearrangements to date and includes patients from three regions of the country. The overall observed rearrangement frequency of 3.44% indicates that rearrangements are relatively uncommon in the admixed population of Brazil.

## Introduction

Approximately 5-10% of all breast cancer diagnoses are associated to germline mutations in highly penetrant cancer predisposition genes. Among these, the tumor suppressor genes *BRCA1* (OMIM # 113705) and *BRCA2* (OMIM # 600185) are the most frequently affected and best studied. The presence of a germline mutation in these genes defines Hereditary Breast and Ovarian Cancer (HBOC) syndrome, an autosomal dominant disorder that predisposes affected individuals to several early-onset tumors including breast, ovarian, prostate, pancreatic cancer and melanoma. Identification of at-risk individuals is important because several risk-reducing strategies can be offered to at-risk patients, especially if they are not yet affected by cancer (ASCO Subcommittee on Genetic Testing for Cancer Susceptibility,1996; Frank *et al.*, 2002; Garber and Offit, 2005; Mai *et al.*, 2009; Allain,2008).

Hundreds of deleterious germline *BRCA1* and *BRCA2* mutations have been described in all populations. These mutations are most frequently single base substitutions (predominantly nonsense mutations) or small frameshift insertions/deletions, which result in premature stop codons and truncated non-functional proteins (http://research.nhgri.nih.gov/bic/) (Ford *et al.*, 1998; Ewald *et al.*, 2009; O'Donnovan and Livingston, 2010). However, in many studies the observed frequencies of deleterious *BRCA1* and *BRCA2* mutations in HBOC families are lower than predicted by linkage analysis or mutation probability models. Pathogenic variations in the coding region or in splice sites of the genes are found in, at most, two thirds of the families carrying *BRCA* mutations (Wera *et al.*, 2003; Linger and Kruk, 2010). Several explanations for this observation have been proposed, including heterogeneous inclusion criteria with different stringencies, the existence of other dominant genes associated with the phenotype, and/or additive effects of multiple lower penetrance alleles. In addition, the presence of pathogenic alterations that escape most of the current gene sequencing-based diagnostic approaches were proposed, including partial or complete exon losses or duplications resulting in an out-of-frame translation and a mutant peptide with abnormal structure and/or function (Petrij-Bosch *et al.*, 1997; Ewald *et al.*, 2009). Several reports confirmed that *BRCA* rearrangements, particularly in *BRCA1*, are indeed quite frequent in HBOC families from selected countries (Preisler-Adams *et al.*, 2006; Hansen *et al.*, 2008; Kang *et al.*, 2010; Ratajska *et al.*, 2008; Stadler *et al.*, 2010; Rudnicka *et al.*, 2013; Pal *et al.*, 2014). These mutations are scattered throughout the gene and although most of them are deletions, duplications and triplications, as well as combined deletion/insertion events have also been described. The higher prevalence of rearrangements in *BRCA1*, compared to *BRCA2*, has been attributed to its molecular structure, which is characterized by an extremely high density of intronic *Alu* repeats and by the presence of a duplicated promoter region containing a pseudogene that favors unequal homologous recombination events (Smith *et al.*, 1996; Puget *et al.*, 2002; Thomassen *et al.*, 2006; Staaf *et al.*, 2008).

The highest proportion of *BRCA1* rearrangements in HBOC families has been observed in The Netherlands, where it represents approximately 36% of the identifiable mutations in the gene in this population (Petrij-Bosch *et al.*, 1997). A similar frequency of deleterious *BRCA1* gene rearrangements has been described in HBOC families from Northern Italy (Montagna *et al.*, 2003), and in Portuguese HBOC families a single founder *BRCA2* rearrangement (c. 156_157insAlu) has been identified in 8% of the families studied (Machado *et al.*, 2007). In contrast, Danish families with HBOC have a *BRCA1* rearrangement prevalence less than 5%, and in Finland and Canada few or no *BRCA1* rearrangements have been identified in high-risk families (Lahti-Domenici *et al.*, 2001; Moisan *et al.*, 2006; Pylkas *et al.*, 2008). Considering the specificity of the mutation prevalence in different populations and the importance of the precise identification of mutation carriers in at-risk families, we aimed to determine the frequency and nature of germline *BRCA1* and *BRCA2* rearrangements in Brazilian HBOC families.

## Patients and Methods

### Patient recruitment

A consecutive sample of 145 unrelated Brazilian patients who were diagnosed with cancer and had a significant personal and/or family history suggestive of HBOC syndrome was evaluated in cancer genetic counseling services from three Brazilian Institutions in the South (Hospital de Clínicas de Porto Alegre, Porto Alegre, RS; n = 69), Southeast (Brazilian National Cancer Institute, INCA, Rio de Janeiro, RJ; n = 43) and Northeast (Laboratory of Molecular Biology and Oncogenetics, Federal University of Bahia, Salvador, BA; n = 33) of the country. The 69 probands from Porto Alegre had been previously studied for the Portuguese founder rearrangement c.156_157insAlu in *BRCA2* (Peixoto *et al.*, 2011).

Cancer-affected probands were approached during their routine clinical visits and invited to participate in the study. None of them had been previously tested for germline *BRCA* mutations, due to restricted access to testing through the public health care system. All participants signed informed consents and fulfilled one or more of the following criteria: (a) personal and family history consistent with the American Society of Clinical Oncology (ASCO) criteria for HBOC syndrome (ASCO Subcommittee on Genetic Testing for Cancer Susceptibility) (ASCO, 1996); or (b) a prior probability for a *BRCA* mutation ≥ 20% using either mutation prevalence tables published by Myriad Genetics Laboratories, Inc. or the Penn II mutation prediction model (Frank *et al.*, 2002; Myriad Genetics). Ethical approval for this study was obtained from the institutional ethics committees of all participating centers.

### Screening for *BRCA* rearrangements by MLPA

Relative copy number quantification of all 24 *BRCA1* and 27 *BRCA2* exons was performed using the SALSA P002B *BRCA1* and SALSA P045 *BRCA2* MLPA probe mix assays (MRC-Holland, Amsterdam, The Netherlands) as recommended by the manufacturer. Multiplex PCR-amplified products were separated by capillary gel electrophoresis in an ABI PRISM 3130XL Genetic Analyzer and analyzed using GeneMapper ID V3.2 software. Information on copy number was extracted with Coffalyser V9.4 Software (MRC-Holland, http://www.mrc-holland.com/). All analyses were performed in duplicate and in at least two independent experiments. Positive results were confirmed in an additional independent experiment performed on a second blood sample. Samples showing *BRCA1* rearrangements identified by the SALSA MLPA P002B kit were then analyzed by a different set of MLPA probes (SALSA P087 MLPA probemix, MRC-Holland, Amsterdam, The Netherlands).

### Characterization of rearrangement breakpoints

To confirm BRCA rearrangements detected by MLPA, all rearrangement-positive samples were submitted to long-range PCR amplification using AmpliTaq Gold® DNA Polymerase (Applied Biosystems, Foster City, USA) and primers specifically designed for the regions of interest. Amplification products of long-range PCR were separated by 2.0% agarose gel electrophoresis and visualized under UV. The mutant (variant size) amplification products were extracted and purified using a Gel Band Purification Kit (Illustra, GE Healthcare UK limited, Buckinghamshire, UK) as described by the manufacturer. Isolated PCR fragments were submitted to bidirectional sequencing using a Big Dye V3.1 Terminator Kit (Applied Biosystems, Foster City, CA, USA) on an ABI Prism 310 Genetic Analyzer (Applied Biosystems, Foster City, USA) with standard protocols. All sequencing electropherograms were analyzed using GeneMapper® Software (Applied Biosystems, Foster City, USA).

### Detection of the c.156_157insAlu *BRCA2* mutation by PCR

To identify the c.156_157insAlu mutation, *BRCA2* exon 3 was PCR amplified and amplicons were visualized by electrophoresis. To confirm the presence of the insertion detected in the first PCR round, a second PCR with specific primers flanking the Alu insertion was performed. All Alu insertion-positive samples were submitted to confirmatory sequencing analysis as described by Teulges *et al.* (2005).

### Statistical Analyses

Sample size was estimated using WINPEPI (PEPI-for-Windows), and SPSS version 18.0 was used for data handling and statistical analyses. For descriptive analysis, categorical variables were described by their absolute frequencies and quantitative variables were expressed as the mean and standard deviation (SD); a significance level of 0.05 was considered acceptable.

## Results

**Figure 1:**
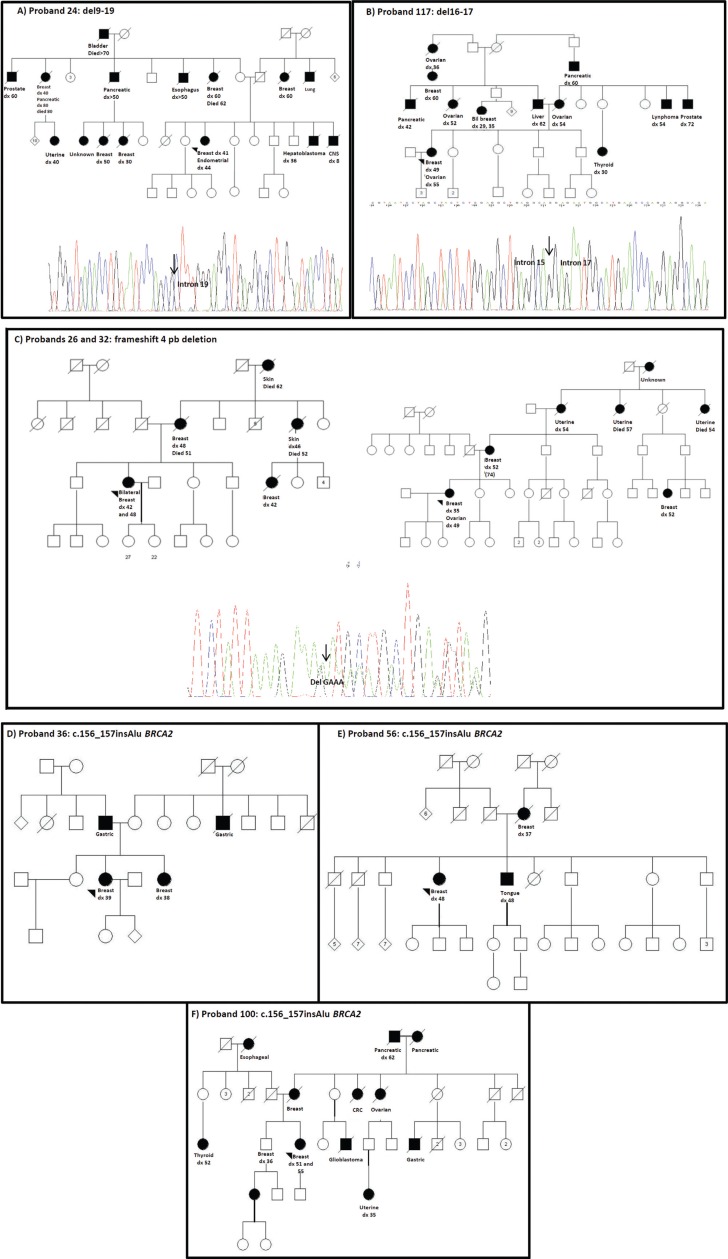
Pedigrees of the mutation carriers. Panels A, B and C: families with germline *BRCA1* mutations identified by MLPA as first mutation screening strategy. Panels D, E and F: families with germline c.156_157insAlu-*BRCA2* mutation identified by PCR.

Clinical data of the 145 unrelated probands included in this study are summarized in Table 1. The mean age at diagnosis of the first HBOC-associated tumor was 43 years, and the most frequent tumor was breast cancer, as expected. Among all included probands, 118 (81.4%) were diagnosed with their first primary tumor before the age of 50 years. The estimated prior probability of carrying a *BRCA* gene mutation was greater than 20% for 65 (44.8%) and 71 (49.0%) probands according to the Myriad mutation prevalence tables and the Penn II model, respectively. Overall, *BRCA* rearrangements were identified in 5 probands (3.4%), with three of them being positive for the Portuguese *BRCA2* founder rearrangement c.156_157insAlu. All *BRCA1-* and one of the *BRCA2*-positive probands had multiple primary tumors. In two cases, positive MLPA results were not confirmed with a second set of MLPA probes, and further testing revealed a point mutation in the MLPA probe hybridization site. Sequencing of the two individuals with unequivocal *BRCA1* rearrangements found by MLPA identified the exact breakpoints*.* The first case (proband 24) had a microdeletion comprising exons 9 to 19, which was visualized after long-range PCR amplification of the flanking regions as a variant amplification product of approximately 450 bp, when compared to the wild-type allele amplification product of 9 kb. Bidirectional sequencing of the variant allele identified the exact breakpoints and characterized this rearrangement as c.547+240_5193+178del, which was previously described (Figure 1A) (Silva *et al.*, 2012). The second case (proband 117) had a microdeletion in exons 16 and 17, which was visualized after long-range PCR amplification of the flanking regions as a variant amplification product of approximately 590 bp when compared to the wild-type allele amplification product of 6 kb. Bidirectional sequencing of the variant allele identified the exact breakpoints and characterized this rearrangement as c.4675+467_5075-990del (Figure 1B). In the other two cases (probands 26 and 32) with a suspected deletion of exon 19, confirmatory MLPA with a second set of probes failed to confirm the presence of a rearrangement. Further sequencing of the region identified the frameshift founder mutation initially described in African Americans (NM_007294.2: c.5177_5180delGAAA) (Figure 1C), which is localized within the sequence corresponding to one of the *BRCA1* exon 19 probes of the SALSA P002B *BRCA1* set (Figure 1). A description of the clinical and family history features of the four *BRCA1* and of the three *BRCA2* germline mutation carriers is summarized in Table 2.

**Table 1:** Clinical characterization of the series (n = 145) of HBOC patients included in this study.

Feature	N	%	Mean (± SD) in years
Gender	144	99.9	
Female			
Age at breast cancer (years)			42.92 (9.5)
			range: 19-69
Breast cancer diagnosed < 50 ys	118	81.4	
HBOC syndrome criteria^1^			
ASCO	84	57.9	
Mutation prevalence^2^ ≥ 20%	65	44.8	
Prior probability^3^ (Penn II) ≥ 20%	71	49.0	
Bilateral breast cancer	18	12.4	
Type of tumor in the proband			
Breast cancer	129	88.9	
Ovarian cancer	6	4.2	
Colorectal cancer	4	2.8	
Other^4^	6	4.1	
Multiple primaries	31	21.4	
≥ 2 Breast	14		
1 breast and 1 ovarian	6		
≥ 2 Breast and 1 ovarian	3		
At least one breast + other	7		
At least one ovarian + other	1		

1 One proband may fulfill more than one criterion

2 Patients with a family history compatible with a mutation prevalence of ≥ 20%

3 Estimated prior probability of being a germline BRCA mutation carrier

4 Gastric cancer, melanoma, carcinoma of the uterine cervix, prostate and kidney cancer.

**Table 2:** Clinical features of the seven probands with germline *BRCA* mutations.

Case #	*BRCA1/BRCA2* mutation identified	Cancer diagnosis (index-case)	Age at diagnosis (1st primary, years)	Cancer family history*	ASCO criteria	Prior Prob. of Mutation in *BRCA* # (%)
24-RS	*BRCA1* Deletion exons 9-19: g.29197_65577 del36381	Multiple primary: breast and endometrial	41	MAT Hepatob (M-36), Esoph (M-N/A), Br (F-30), Panc (M-N/A), Blad (M-N/A), Br (F-50), Br and Panc (F-40,80),Prost (M-60), Ut (F-40), CNS (M-8), Br (F-60).	Yes	30.1
				PAT Br (F-60), Lu (M-N/A)	No	6.9
117-BA	*BRCA1* Deletion exons 16-17 c.4675+467_5075-990del	Multiple primary: breast and ovarian	49	MAT Ovarian (F-54), Thyr (F-30), Lymph (M -54) and Prost (M-72).	Yes	79.0
				PAT Liv = (M 62), Ovarian (F52), Ovarian (F 60), Panc (M-42), Bilateral Br (F-29,35), Br (F 60), Panc (M 60).		
32-RS	*BRCA1* 5296del4	Multiple primary: breast and ovarian	35	MAT Br (F-52), Ut (F-54), Ut (F-47) Ut (F-N/I), Br (F-52)	Yes	39.1
36-RS	*BRCA1* 5296del4	Multiple primary: bilateral Breast	46	MAT Br (F-48), Br (F-42),Skin (F-46), Skin (F-N/A)	Yes	30.1
100-RS	*BRCA2* c.156_157insAlu	Multiple primary: bilateral Breast	51	MAT Br (F-62), CCR (M-80), Ut (F-35), Ovarian (F-45), gastric (M-52),panc (M- 62), panc (F- 67), Glioblast (M-38)	Yes	10.6
36-RJ	*BRCA2* c.156_157insAlu	Breast	39	MAT Br (F-36), Gastric (M-N/A)	Yes	15.8
56-RJ	*BRCA2* c.156_157insAlu	Breast	48	MAT Br (F-37), Tongue (M-45), Yes	15.8	

Legend: RS = family recruited from Rio Grande do Sul; BA = family recruited from Salvador -BA.MAT = cancer history in the maternal side of the family, PAT = cancer history in the paternal side of the family;

* Other cancer diagnoses in family are indicated by the abbreviated cancer type (Br = breast, Prost = prostate; Esoph = esophageal; Hepatob = hepatoblastoma; End= endometrial; CNS = central nervous system, Panc = pancreatic, Blad = bladder; Thyr =Thyroid; Lymph = Lymphoma; Glioblast = glioblastoma, Ut = uterine cancer, not defined whether cervix or endometrium) followed by sex (M = male, F = female) and age at diagnosis (N/A= not available).

# according to Myriad mutation prevalence tables.

## Discussion

Using a combined strategy of MLPA and targeted c.156_157insAlu BRCA2 rearrangement testing as a first approach to screen for *BRCA1/BRCA2* germline mutations in a series of Brazilian HBOC patients, we identified seven (4.82%) mutation carriers (with point mutations explaining the abnormal initial MLPA finding in two of them). *BRCA1* rearrangements are usually more prevalent than those in *BRCA2* mostly due to the high density of *Alu* elements throughout the *BRCA1* locus, which seem to be particularly frequent in certain populations. In addition to possible founder effects in specific populations, rearrangements have been most commonly encountered in probands and families with multiple primary cancer diagnoses in at least one individual (Gad *et al.*, 2002; Montagna *et al.*, 2003; Pavlicek *et al.*, 2004; Mazoyer, 2005; Woodward *et al.*, 2005; Walsh *et al.*, 2006). This phenotype was also observed in the majority of rearrangement-positive patients from the current series, reinforcing that rearrangements should always be looked for in families where at least one cancer-affected individual has more than one primary tumor.

The *BRCA1* deletion 9-19, identified in a proband that developed breast cancer at the age of 41 and endometrial cancer at the age of 44 years has been described in an Italian patient (Montagna *et al.*, 2003; Silva *et al.*, 2012). We were able to trace the family history back to the probands maternal grandfather, who emigrated to Brazil from Italy in the 19^th^ century. On the other hand, deletions involving exons 16 or 17 are quite common and have been described in several populations. However, a rearrangement involving breakpoints at Alu regions in intron 15 and intron 17 has not been described to our knowledge (Santarosa *et al.*, 1999; Hartmann *et al.*, 2004; Rodríguez *et al.*, 2010).

An interesting result from this mutation screening strategy was the identification by MLPA of a small frameshift mutation (a deletion of four nucleotides in *BRCA1* exon 19, c.5177_5180delGAAA) in two families. Since the mutation occurs within the region complementary to the exon 19 probe, hybridization did not occur and a call for an exon 19 deletion was made. The use of a second MLPA kit with a different probe for that specific region failed to identify the rearrangement, and sequencing through the region confirmed the frameshift mutation. This illustrates the importance of always confirming MLPA results with an alternative mutation detection method in the diagnostic setting. Interestingly, this particular frameshift mutation has been previously described as a founder mutation in African Americans and has been associated with more aggressive tumors, diagnosed at younger ages. Both of the mutation-positive families identified in our study reported European ancestry (German), and although the probands have been diagnosed with multiple primary tumors, there is no evidence in either of them for a more aggressive clinical course (Qing *et al.*, 1997, 2000; Olopade *et al.*, 2003; Ferla *et al.*, 2007).

Considering the existence of a founder *BRCA2* rearrangement (c.156_157insAlu), which is very common in Portugal, we added a second screening protocol specific for this particular mutation in this investigation. This strategy enabled the identification of the founder in three families, which is not unexpected given the high proportion of Portuguese descendants among the Brazilian population (Marrero *et al.*, 2005). This rearrangement had not been previously reported in southern and northeastern Brazil, but has been seen in HBOC families from the southeastern region of the country (Peixoto *et al.*, 2011, Félix *et al.*, 2014). Our results reinforce the importance of characterizing mutations in specific populations.

Most of the studies describing the prevalence of *BRCA1* rearrangements in HBOC individuals have screened for such mutations only after a negative result in a full gene sequencing approach. Considering the cost and complexity of sequencing the entire coding region of both *BRCA1* and *BRCA2* genes, we designed this study to verify whether MLPA and a specific protocol for a founder *BRCA2* mutation could be an effective strategy as an initial mutation screening approach. Although we did identify germline *BRCA1* mutations and the Portuguese founder mutation in this series, the mutation frequency was relatively low, and in the majority of the patients included the molecular diagnosis remains undetermined. Thus, we conclude that MLPA can be used as an initial approach for screening *BRCA1* mutations in HBOC families, especially considering that it is an inexpensive and straightforward methodology which enables mutation screening of the coding region within a few hours. In populations where a known founder mutation occurs, screening for this specific founder as an initial step can be even more effective. However, we emphasize that this investigation is only partial and that continued investigation by full gene sequencing must be proposed in all high-risk families if such an initial approach is chosen and renders negative results.

In conclusion, this study comprises the largest Brazilian series of HBOC families tested for *BRCA1* and *BRCA2* rearrangements to date, and includes patients from three regions of the country. Although this series cannot be considered representative of the entire Brazilian population, the overall observed rearrangement frequency of less than 5% suggests that BRCA rearrangements are relatively uncommon in this heterogeneous population.
